# Contemporary approaches to visual prostheses

**DOI:** 10.1186/s40779-019-0206-9

**Published:** 2019-06-05

**Authors:** Rebecca M. Mirochnik, John S. Pezaris

**Affiliations:** 1 Department of Bioengineering, Northeastern University, Boston, MA USA; 2 Department of Neurosurgery, Massachusetts General Hospital, Boston, MA USA; 3 Department of Neurosurgery, Harvard Medical School, Boston, MA USA

**Keywords:** Visual prosthesis, Blindness, Artificial vision

## Abstract

Visual prostheses serve to restore visual function following acquired blindness. Acquired blindness (as opposed to congenital blindness) has many causes, including diseases such as retinitis pigmentosa, glaucoma, and macular degeneration, or trauma such as caused by automobile accident or blast damage from explosions. Many of the blindness-causing diseases target the retina or other ocular structure. Often, despite the loss of sensitivity to light, the remainder of the visual pathway is still functional, enabling electrical devices to deliver effective and meaningful visual information to the brain via arrays of electrodes. These arrays can be placed in any part of the early visual pathway, such as the retina, optic nerve, lateral geniculate nucleus, or visual cortex. A camera or other imaging source is used to drive electrical stimulation of remaining healthy cells or structures to create artificial vision and provide restoration of function. In this review, each approach to visual prostheses is described, including advantages and disadvantages as well as assessments of the current state of the art. Most of the work to-date has been targeting stimulation of (a) the retina, with three devices approved for general use and two more in clinical testing; (b) the lateral geniculate nucleus, with efforts still in the pre-clinical stage; and (c) the cortex, with three devices in clinical testing and none currently approved for general use despite the longest history of investigation of the three major approaches. Each class of device has different medical indications, and different levels of invasiveness required for implantation. All contemporary devices deliver relatively poor vision. There has been remarkable progress since the first proof-of-concept demonstration that used stimulation of the primary visual cortex, with the field exploring all viable options for restoration of function. Much of the progress has been recent, driven by advances in microelectronics and biocompatibility. With three devices currently approved for general use in various parts of the world, and a handful of additional devices well along in the pipeline toward approval, prospects for wide deployment of a device-based therapy to treat acquired blindness are good.

## Background

Visual prostheses are devices intended to restore lost visual function via the use of electronic circuitry and electrical impulses. With an estimated 36 million and growing blind individuals, the significance of a cure for blindness is clear and increasing [1]. Visual prostheses can provide benefit to those with severe vision loss, especially if no other medical treatment options exist.

Experimental work in visual prosthetics started in earnest in the early 1900s and has grown since then (excellent historical perspectives have been written by Donaldson and Brindley [2], and Lewis and Rosenfeld [3]). In 1929, Foerster [4], quickly followed by Krause and Schum in 1931 [5] stimulated brain areas at the occipital pole, creating *phosphenes* — small, electrically-evoked visual percepts. These observations were amplified by Button and Putnam [6] who demonstrated independent, punctate phosphenes through multiple channels of cortical stimulation. Brindley and Lewin then embarked on a series of experiments that included a ground-breaking report in 1968 [7] describing the first implant to generate sufficiently many phosphenes to convey visually-based patterns [8, 9]. Brindley and Lewin’s seminal work, along with advances in technological miniaturization and biocompatibility, and parallel efforts to treat Parkinson’s disease, chronic pain, and hearing loss [10–12], led to an acceleration of research through the last few decades that has expanded the number of efforts to the present dozens of groups working world-wide on visual prosthesis devices.

**Fig. 1:**
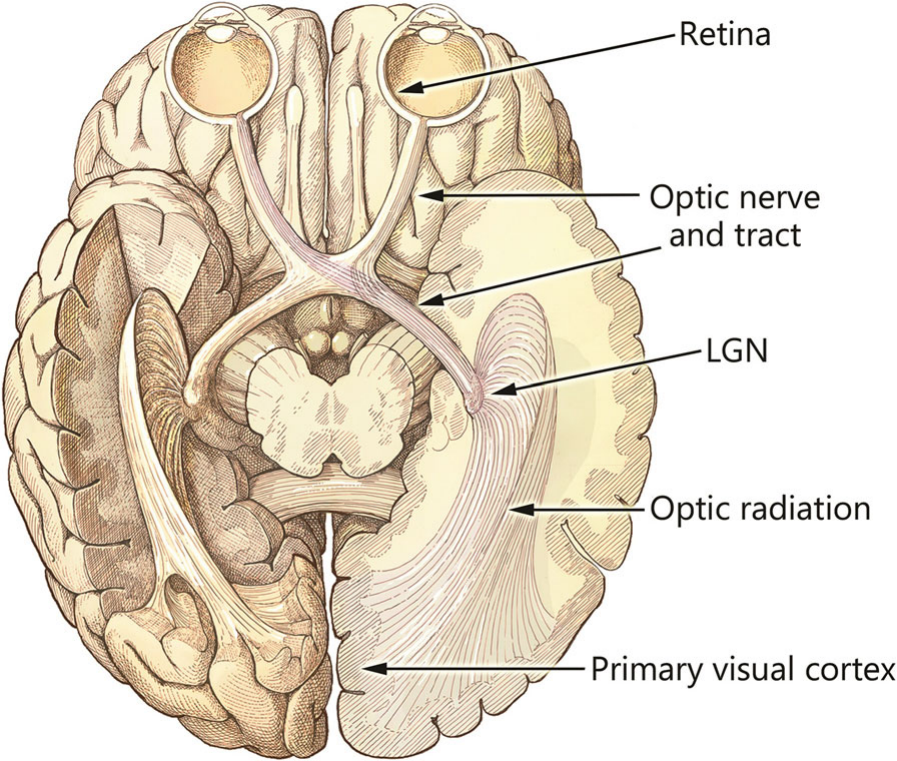
A ventral view of the human brain showing the early visual pathway. Normally, light enters the system through the optics of the eyes, and is focused on the retina where photons are converted to neural activity. From the retina, this activity flows down the optic nerves and through the chiasm, along the optic tract to the lateral geniculate nucleus of the thalamus (LGN). The chiasm serves to sort fibers from the two eyes such that signals are combined by visual hemifield (both right hemifields go to the left LGN, and vice-versa). From the LGN, signals then flow through the optic radiation to the primary visual cortex (V1), and on to the higher visual areas

Normal function of the visual pathway (Fig. 1) begins with light entering the eye and being converted into neural signals by the photoreceptors of the retina. These neural signals are then sent by the retinal ganglion cells along the optic nerve formed by their axons to the lateral geniculate nucleus (LGN). From the LGN, signals propagate along the optic radiation to the primary visual cortex (V1). From V1, the central visual signal path begins to fan out quickly to areas of the brain that specialize in visual function.

Depending on the etiology of blindness, a variety of anatomically-targeted approaches along the visual pathway are viable. For example, retinitis pigmentosa (RP) causes degeneration of the rods and cones that constitute the primary photosensitive cells in the retina, although the inner nuclear layer and retinal ganglion cell layer are unaffected until the disease reaches later stages [13]. Therefore, stimulation of the remaining healthy retina is an option; this option is especially compelling given that no other treatments for late-stage RP currently exist [13]. However, if the retina has degenerated beyond possible stimulation or if damage has occurred further down the visual pathway, such as in glaucoma that affects the optic nerve, the remainder of the visual pathway is frequently intact and viable, allowing prosthetic stimulation to be successfully applied later along the visual stream [14]. Currently, the majority of visual prosthesis research has centered on the retina, with other, non-retinal approaches remaining somewhat more experimental.

Besides current technological approaches to cure blindness, several biological approaches exist, although each comes with both benefits and disadvantages. Gene therapy has focused on loss of function mutations in which introducing a wild-type gene can potentially restore function [15]. This approach has been successful in clinical preservation or partial restoration of vision in Leber’s congenital amaurosis [16, 17] and choroderemia [18]. However, gene therapy is limited to recessive mutations [15]. Optogenetic approaches aim to activate residual retinal cells by transfection with viral carriers that express light-sensitive cell-membrane ion channels and thus confer phososensitivity. Although converted cells still require a high level of light and a successful intervention requires a reasonably large population of surviving retinal cells [19, 20], efficacy has been shown in a primate model [21, 22] and clinical trials are ongoing for treating retinitis pigmentosa (clinicaltrials.gov registration numbers NCT02556736, NCT03326336). Both of these approaches require unaffected retinal tissue and are thus most applicable to earlier stages of retinal degenerative diseases prior to the extensive retinal remodeling that is observed in advanced stages [23, 24].

## Overview

The following paragraphs will describe the current approaches to visual prosthesis devices, organized by location of approach and following along the natural flow of information in the early visual pathway: retina, optic nerve, lateral geniculate nucleus (LGN), and visual cortex (see Fig.1; to the best of our knowledge, there is no current effort attempting sight restoration using the optic radiation that projects from the lateral geniculate nucleus to visual cortex). The advantages and disadvantages of each approach will be described, as well as the current research being conducted and clinical testing or deployment if any. Important previous reports upon which we build here include the excellent compendium “Artificial Vision” edited by Gabel [25], and review articles by Weiland *et al*. [26], Fernandes *et al*. [27], and Lewis *et al*. [28]. Our present contribution to the field is to include a more detailed look at the thalamic approach, and to guide the reader toward projects that currently have ongoing clinical trials.

## Visual prostheses

### Retina

The retina is a major focus for visual prostheses. Various structures of the retina can be targeted for electrical stimulation, including the outer layer of light-sensitive photoreceptor cells, inner layer of bipolar cells, and the layer of retinal ganglion cells whose axons form the optic nerve [29]. Photoreceptor cells consist of cone cells that support high-acuity color vision, and rod cells that support low light level vision. By simplification, photoreceptors synapse with bipolar cells which subsequently synapse with retinal ganglion cells. The retina is frequently chosen as a visual prosthesis target for a number of reasons: its extracranial location allows easy surgical access, and an implant in a single eye can potentially cover nearly the entire visual field [30]. The retina's simpler architectual organization as compared to locations further down the visual stream is appealing as well [31]. Additionally, even with advanced degeneration, sufficiently many retinal neurons are frequently still present and capable of generating signals to convey images [32].

However, the retinal approach has several disadvantages. The retina is delicate and has varying availability across its extent, which can restrict electrode count, field of view, and subsequent visual acuity [31, 32]. Retinal diseases may lead to retinal reorganization, complicating known retinal maps, stimulation parameters, and evoked percepts [29]. Furthermore, successful retinal implants require a significant number of remaining retinal ganglion cells, limiting scope and applicability [33]. Ailments affecting later points of the visual stream, including glaucoma or traumatic injuries, cannot be aided with such prostheses [34]. For degenerative retinal diseases whose progression can affect the number of retinal cells remaining, prosthesis designers need to take retinal state into account to achieve success [35].

Retinal prostheses frequently follow one of two formats: (1) an electrode array driven by an external camera via a visual processing unit stimulates retinal tissue or (2) an intraocular photodiode array converts light energy into electrical signals to activate nearby retinal cells [35–43]. Typical *epiretinal* prosthesis designs such as the Argus II, have an electrode array implanted on the inner surface of the retina, adjacent to the vitreous humor, targeting the retinal ganglion cell layer. Usually epiretinal prostheses have an external imaging source, such as a miniature video camera mounted into a set of goggles. Typical *subretinal* prosthesis designs have an electrode array implanted on the outer layer of the retina and target adjacent bipolar cells. Often subretinal prostheses integrate the imaging sensor with the electrode array, such as the Alpha AMS, with the intention of replacing failed photoreceptor cells with photodiodes. Typical *suprachoroidal* prosthesis designs have an electrode array implanted between the choroid and sclera or on the sclera and target retinal ganglion cells. They also typically use an external camera similar to the epiretinal prosthesis. The following sections describe each approach and its benefits and challenges in further detail.

#### Epiretinal prosthesis

An epiretinal prosthesis is implanted on the surface of the retina at the vitreous cavity, often secured with a tack, and transmits information from a camera to stimulate the retinal ganglion cells [35]. Its proximity to the retina allows for a low stimulation threshold which in turn allows for a smaller size, while contact with the vitreous cavity fluids helps dissipate heat from the device [35, 44]. While having a processing unit between the camera and the simulating array can allow for substantial image analysis and manipulation, such systems typically require head movement from the user to steer the camera, not the more natural eye-and-head combination [31, 34, 45]. The conventional use of a tack to anchor the electrode array may also be considered a disadvantage, as it possibly causes retinal damage and long-term mechanical stability issues [34]. Finally, the retinal ganglion cell stimulation must be highly targeted to the cell bodies to avoid unintended activation of fibers originating from other parts of the retina which may pass under the electrodes [46]. While initial work focused on feasibility, especially with retinitis pigmentosa patients, the epiretinal approach has advanced the farthest within the field, with devices receiving governmental approval for clinical use in both the United States and Europe [46, 47]. Limitations of the epiretinal approach include acuity that is quite low compared to normal vision [35], difficulty changing phosphene color [48], and, importantly, a field of vision restricted to the typically small span of the electrode array [49]. As we will see below, subretinal devices often share many of these factors.

Currently, the only visual prosthesis to have received both United States Food and Drug Administration (FDA) and European Commission (CE) approval, both for use against Retinitis Pigmentosa, is Second Sight’s Argus II: a 60-electrode epiretinal array receiving visual information wirelessly from an external camera mounted on a set of glasses [35–37, 50]. The Argus II has enabled patients to read, albeit slowly, recognize words, and detect motion, with a maximum visual acuity of 20/1260 over a highly-limited visual field [35, 37, 50]. Device safety has been evaluated, and the device appears to be well tolerated and safe. In a five year trial, 60% of patients had no serious adverse events, although common but less serious effects included conjunctival erosion and ocular hypotony [36]. One retinal detachment, a frequent concern with retinal implants, was recorded [36]. While the Argus II is the most established epiretinal device, others are not far behind in development. For example, the IRIS 2 [51] carries a CE mark for use with outer retinal degeneration, and is being tested in an ongoing clincal trial for additional indications (NCT02670980), along with the EPIRET3 that has been successfully implanted and well tolerated in RP patients [52, 53]. We expect to see additional progress for epiretinal efforts in the near future.

#### Subretinal prosthesis

With an array that combines electronic photosensors and stimulating contacts and is positioned on the outer retina, subretinal prostheses are designed to replace lost or malfunctioning photoreceptors with photodiodes, an attractive method given that even in highly degenerated retinas, neural activity can be evoked by prosthetic electrical stimulation [38]. The subretinal location of the photosensitive aspect of the array enables such devices to take advantage of both natural eye movements and retinal circuitry, and recipients can use a subretinal prosthesis with little learning effort [54]. Additionally, intimate proximity of the stimulating contacts to the retinal circuitry allows for lower stimulation thresholds than with other approaches [44]. The limited available space, while restricting implant thickness and power, allows subretinal prostheses to be held in place without a tack [29, 54]. Further constraints of the subretinal approach that are largely shared with the epiretinal approach include the limited possible visual acuity using photodiodes (an expected maximum of Snellen 20/250 due to limitations based on current spread), the lack of color perception, and the limited field of vision, which is constrained to only the extent of the array as it serves as both imaging device and stimulator [40, 41]. Typical designs, such as the Alpha AMS which is described in more detail below, are sensitive to and image natural light directly, eliminating the need for an external camera; the current sole exception is the PRIMA device [55] that uses infrared-sensitive photodiodes and encodes the visual scene from an external camera into infra-red images to drive its array. Having an external camera and image processing, such as used with typical epiretinal implants, creates a significant functional advantage for the PRIMA device over other subretinal devices. With the Alpha AMS, for example, image processing is limited by the room for local circuitry on the implant itself, and currently supports only basic contrast and gain control [40, 41].

Similar to the epiretinal approach, one subretinal series of devices has received governmental approval in Europe: Retina Implant AG’s Alpha IMS and its successor the Alpha AMS. Given that the AMS has replaced the IMS for clinical purposes (including extension of the original CE mark to the new device), we will concentrate on the newer, and largely improved AMS for this report [42]. For a comparative technical evaluation of the two devices, see, for example the report by Daschner et al. [43]. The Alpha AMS consists of an array of 1600 photodiodes that is implanted in the outer retina. The photodiodes convert light to current that is used to stimulate adjacent bipolar cells [39–42, 56]. Implanted patients have been able to read letters, combine letters into words, perceive and localize light, detect motion, navigate familiar locations, and identify and grasp objects [39, 42, 56]. The highest visual acuity measured was Snellen 20/546, an important improvement compared to both epiretinal and suprachoroidal prostheses, as well as current non-retinal devices [21, 24, 25, 34]. The Alpha IMS and AMS devices have shown similar adverse effects as the epiretinal Argus II, with the most common events being elevated intraocular pressure (IOP), conjuctival erosion, and retinal detachment; each of these events were associated with device implantation and removal respectively and all adverse events were successfully resolved [42, 57]. IOP was seen shortly after device implantation in one case, and retinal detachment was associated with device removal in one case as well.

As mentioned above, an important subretinal prosthesis currently undergoing clinical testing is the PRIMA device (NCT03333954) [55, 58]. This design uses an array of photodiodes that convert varying intensities of infra-red illumination to localized electrical stimulation of retinal tissue. Externally-worn goggles amplify the video stream from a conventional camera into bright infrared illumination that is focused by the optics of the eye onto the implanted array. This hybrid approach of translating the external visual field into an infrared image, potentially modifying it along the way, delivered with sufficient brightness to power the local circuitry at each photosensitive cell, has substantial promise. Along with the Alpha AMS, it remains one of the few retinal approaches that supports visual exploration through normal eye movements.

#### Suprachoroidal prosthesis

Finally, the *suprachoidal* retinal prosthesis is implanted between the choroid and sclera, or in the scleral pocket for the *suprachoroidal-transretinal* variant, and stimulates retinal neurons. The position of the suprachoidal prosthesis intends to avoid retinal damage that can be caused by direct contact [29]. The scleral pocket provides mechanical stability, while the choroid blood vessels aid in heat dissipation [33, 44]. In addition, the retinally-removed location facilitates a less challenging surgery than other visual prostheses [44]. While beneficial in some respects, the distance from the retina requires suprachoroidal prostheses to stimulate through the higher electrical resistance of the retinal pigment epithelium, resulting in higher stimulation thresholds which can increase the risk of damage [59]. Furthermore, the higher thresholds and subsequently higher charge injection requirements increase the impact of current spreading, which results in decreased visual contrast and resolution [33].

Currently, no suprachoroidal prostheses have been approved beyond Phase 1 clinical trials [33, 60]. Fujikado et al. [33] implanted a suprachoroidal-transretinal prosthesis in several RP patients, eliciting percepts with no serious adverse events. In addition, Ayton and colleagues successfully implanted a device that allowed late-stage RP patients to localize light and recognize letters, although with an optotype acuity of approximately 20/8000 [60].

### Optic nerve

If intact, the optic nerve is an interesting potential target for visual replacement: the optic nerve has an extracranial segment and thus can be accessed with minimally invasive surgery, and would support a full field view despite a high level of retinal cell disease [30, 61]. Evoked potentials via the optic nerve have been shown to have the same wave shape as normal visual potentials, further providing support for the optic nerve, and prior reports have described a decreasing stimulation threshold over long periods of device implantation [62, 63]. Still, optic nerve stimulation has thus far been demonstrated with only low resolution and low apparent brightness, requiring high stimulation to evoke percepts, and while the optic nerve approach is attractive in part due to a potentially simple surgical implantation, it still requires an active nerve [61, 62]. Optic nerve prostheses can take two forms: cuff electrodes and penetrating electrodes [61, 62, 64–68]. Current optic nerve researchers have generated phosphenes in RP patients, as well as demonstrated object localization and identification, albeit with very long recognition times [61, 65].

### Lateral geniculate nucleus

The lateral geniculate nucleus is a visual relay center within the thalamus, receiving input from the optic nerve and forwarding information to the visual cortex. The LGN has become an increasingly attractive target to researchers given that advances in deep brain stimulation have created easy surgical access to the thalamus [69]. Additionally, the compact structure of the LGN supports a wide prosthetic visual field, and the overrepresentation of the fovea is thought to allow for higher acuity vision than other approaches [30, 70]. Additional anatomical benefits include the simple and well characterized receptive fields and the separated visual subdivisions of the LGN that may support color artificial vision [69, 70]. Full visual field coverage would require two separate arrays, one in each hemisphere, as the LGN is post optic-chiasm and like all subsequent visual areas, each hemisphere represents only one half of the visual field [30]. Current LGN research has affirmed that stimulation can produce phosphenes and that such stimulation produces similar responses in the visual cortex as natural visual stimulation [69, 70].

### Visual cortex

The visual cortex was among the first locations considered for a visual prosthesis, with seminal work by Brindley and Lewin inducing phosphenes in the late 1960s [7]. In addition, the first complete visual prosthesis was a cortical device, developed by Dobelle and colleagues some 30 years later [71]. Since then, substantial additional effort has focused on the visual cortex due to its large surface area, straightforward stimulation procedure, and applicability to all forms of blindness other than cortical injury or stroke [3, 28]. The use of penetrating intracortical rather than surface stimulation has enabled researchers to stimulate at greatly lower current levels than expected, as well as allowing for closely-spaced electrodes [72]. However, similar to the retina, the cortex may experience reorganization after blindness, complicating phosphene mapping, and, similar to many other prostheses, current designs for cortical prosthesis rely on head-steering of the scene camera and do not make use of eye movement [28, 73]. Most recently, Second Sight, developers of the Argus II retinal prosthesis, have received conditional FDA approval for clinical trials of the Orion Cortical Visual Prosthesis System [74] (NCT03344848), the Universidad Miguel Hernandez de Elche in Spain has begun a clinical study on the CORTIVIS device (NCT02983370), and UCLA is running a clinical study on the NeuroPace RNS System (NCT02747589). Several other groups have cortical prostheses in various states of development as well [75–79].

## Clincal trials

The current list of clinical trials that have registered with the US National Library of Medicine (clinicaltrials.gov) for visual prosthesis devices is found in Table 1. As can be seen from the list there are a small number of efforts that have advanced to the stage of clinical trial with their device. The number of research groups working on devices that have not yet advanced to the clinical stage is substantially larger, with some two dozen currently known (see, for example, the list at http://www.eye-tuebingen.de/zrenner/retimplantlist/). Note that the list in Table 1 excludes drug-based interventions as mentioned earlier, as well as non-invasive sensory-substitution devices like BrainPort [80] and The vOICe [81].

**Table 1:** Current clinical trials of visual prosthesis devices

Type	Clinical trial	Title/Sponsor	Status	Disease	Device	Last update
Epiretinal	NCT00407602	Argus II Retinal Stimulation System Feasibility Protocol *Second Sight Medical Products*	A	RP	Argus II	2015-05-29
	NCT01490827	Argus II Retinal Prosthesis System Post-Market Surveillance Study *Second Sight Medical Products*	R	oRD RP	Argus II	2017-10-09
	NCT01860092	New Enrollment Post-Approval Study of the Argus II Retinal Prosthesis *Second Sight Medical Products*	R	RP	Argus II	2018-07-23
	NCT01999049	Observational Study of the Argus II Retinal Prosthesis System *University Health Network, Toronto*	U	RP	Argus II	2015-04-24
	NCT02227498	Argus II Retinal Prosthesis System Dry AMD Feasibility Study Protocol *Second Sight Medical Products*	A	AMD	Argus II	2017-10-09
	NCT02303288	Post-Market Study of the Argus II Retinal Prosthesis System – France *Second Sight Medical Products*	A	RP C	Argus II	2018-07-20
	NCT03248388	Argus II/ORCAM Device Study *Mayo Clinic, Second Sight Medical Products, Orcam Technologies Ltd.*	R	RP	Argus II ORCAM	2018-11-12
	NCT03418116	Argus II Retinal Prosthesis System -- Better Vision RP Study *Second Sight Medical Products*	R	RP C	Argus II	2017-09-05
	NCT03510234	Self-confidence Study in Patients With Argus II Artificial Retina *University Hospital, Strasbourg*	R	RP	Argus II	2018-07-06
	NCT03635645	Experimental and Clinical Studies of Retinal Stimulation *University of Michigan*	R	RP	Argus II	2018-08-17
Subretinal	NCT02670980	Compensation for Blindness With the Intelligent Retinal Implant System (IRIS 2) in Patients With Retinal Dystrophy *Pixium Vision SA*	A	RP CRD C	IRIS 2	2017-01-24
	NCT03333954	Feasibility Study of Compensation for Blindness with the PRIMA System in Patients With Dry Age Related Macular Degeneration *Pixium Vision SA*	A	dAMD	PRIMA	2018-07-12
	NCT03392324	PRIMA Feasibility Study in Atrophic Dry AMD *Pixium Vision SA*	R	dAMD	PRIMA	2018-05-01
	NCT03561922	Impact on Daily Life of Patients Using the Subretinal Implant Alpha AMS *Retina Implant AG*	R	RD	Alpha AMS	2018-10-26
	NCT03629899	Retina Implant Alpha AMS in Blind Patients With Retinitis Pigmentosa *Wills Eye/Retina Implant AG*	R	RP	Alpha AMS	2019-01-09
Choroidal	NCT03406416	Study of a Suprachoroidal Retinal Prosthesis *Mobius Medical Pty Ltd., Bionic Vision Technologies, and four others*	E	RP C	Bionic Eye	2018-03-22
Cortical	NCT02747589	Feasibility of Stimulating the Visual Cortex in Blind *University of California, Los Angeles*	A	Ba	NeuroPace	2018-02-12
	NCT02983370	Development of a Cortical Visual Neuroprosthesis for the Blind *Universidad Miguel Hernandez de Elche, Hospital IMED Elche*	R	B	CORTIVIS	2017-10-27
	NCT03344848	Early Feasibility Study of the Orion Visual Cortical Prosthesis System *Second Sight Medical Products*	R	Ba	Orion	2018-07-16

Table 1 shows the current status of clinical trials for visual prostheses that have registered with the US National Library of Medicine (clinicaltrials.gov), organized by device type. Completed trials are not shown. For each trial, the title and sponsors are given, along with the current status (A – Active, not recruiting; E – Enrolling by invitation; R – Recruiting; U – Status unknown), the indicated disease (AMD – Age-related Macular Deveneration; B – Blindness; Ba – Blindness, acquired; C – Choroidermia; CRD – Cone Rod Dystrophy; dAMD – Dry Age-related Macular Degeneration; oRD – Outer Retinal Degeneration; RD – Inherited Retinal Dystrophy; RP – Retinitis Pigmentosa), the device associated with the clinical trial, and the date of the most recent update posted to clinicaltrials.gov.

## Conclusions and future directions

The field of visual prostheses has grown rapidly in the recent years, from proof-of-principle demonstrations that generated simple percepts in 1968 to multiple devices granted FDA and CE approval for clinical use [7, 36, 41, 51]. Research has confirmed that several structures of the early visual pathway are viable as targets to restore vision: retina, optic nerve, LGN, and visual cortex. While each approach comes with advantages and disadvantages, research on the retinal approach has advanced the farthest.

As previously described, the retinal prostheses can be separated into three varied approaches: epiretinal, subretinal, and suprachoroidal. The epiretinal approach places stimulation arrays on the inner limiting membrane, close to the retinal ganglion cells, allowing for low electrical thresholds but at the cost of potential retinal damage and off-target stimulation. The subretinal approach places imaging and stimulating arrays within the thin sheet of retinal tissue, allowing such devices to have reduced stimulation thresholds and to take advantage of natural eye movements and any remaining retinal circuitry. However, practical concerns about the extent of implants within the ocular structure limit the extent of the visual field that currently can be stimulated, and the retinal architecture may place an ultimate limit on visual acuity for both epi-and sub-retinal approaches that is not present for thalamic and cortical approaches. The suprachoroidal approach avoids retinal damage and visual field extent constraints by positioning stimulating electrodes outside of the retina, in a scleral pocket, but requires higher levels of stimulation to achieve similar effects and has inherently poorer potential resolution.

Devices targeting the different stages of the early pathway have been variously shown to improve light detection, character recognition, and mobility, although future work clearly remains to progress beyond the very crude level of vision afforded by current devices. Specifically, determining the optimal prosthesis location and further developing prosthesis technology would immensely advance the field and provide patient benefit. Given that each approach has its advantages and weaknesses, including appropriateness for disease stage and etiology, device choice must be carefully considered to best enhance vision in patients. Efforts to address electrode count and spacing, to sharpen visual acuity and expand visual field area, as well as size constraints, power constraints, and external image processing will advance the quality of artificial vision for recipients. Overall, despite current limitations in resolution and applicable disease conditions, visual prostheses have shown great potential for positive impact on patient lives, and this potential will only increase with additional research and development.

## Abbreviations

CE Mark: Aapproval for drugs or devices given by the European Commission
CE: European Commission
FDA: United States Food and Drug Administration
IOP: Intraocular pressure
LGN: Lateral geniculate nucleus of the thalamus
RGC: Retina ganglion cell
RP: Retinitis pigmentosa
V1: Primary visual cortex
